# The plant secondary compound swainsonine reshapes gut microbiota in plateau pikas (*Ochotona curzoniae*)

**DOI:** 10.1007/s00253-021-11478-6

**Published:** 2021-08-17

**Authors:** Shien Ren, Chao Fan, Liangzhi Zhang, Xianjiang Tang, Haibo Fu, Chuanfa Liu, Shangang Jia, Yanming Zhang

**Affiliations:** 1 Key Laboratory of Adaptation and Evolution of Plateau Biota, Northwest Institute of Plateau Biology, Chinese Academy of Sciences, Xining, 810008 China; 2 Qinghai Provincial Key Laboratory of Animal Ecological Genomics, Xining, 810008 China; 3 University of Chinese Academy of Sciences, Beijing, 100049 China; 4 Key Laboratory of Restoration Ecology of Cold Area in Qinghai Province, Northwest Institute of Plateau Biology, Chinese Academy of Sciences, Xining, 810008 China; 5 College of Grassland Science and Technology, China Agricultural University, Beijing, 100193 China

**Keywords:** Gut microbiota, 16S rDNA, Plant secondary compounds, Swainsonine, Plateau pika (*Ochotona curzoniae*)

## Abstract

**Abstract:**

Plants produce various plant secondary compounds (PSCs) to deter the foraging of herbivorous mammals. However, little is known about whether PSCs can reshape gut microbiota and promote gut homeostasis of hosts. Using 16S rDNA sequencing to investigate the effects of PSCs on the gut microbiota of small herbivorous mammals, we studied plateau pikas (*Ochotona curzoniae*) fed diets containing swainsonine (SW) extracted from *Oxytropis ochrocephala*. Our results showed that both long- and short-term treatment of a single artificial diet in the laboratory significantly reduced alpha diversity and significantly affected beta diversity, core bacteria abundance, and bacterial functions in pikas. After SW was added to the artificial diet, the alpha diversity significantly increased in the long-term treatment, and core bacteria (e.g., *Akkermansiaceae*) with altered relative abundances in the two treatments showed no significant difference compared with pikas in the wild. The complexity of the co-occurrence network structure was reduced in the artificial diet, but it increased after SW was added in both treatments. Further, the abundances of bacteria related to altered alanine, aspartate, and glutamate metabolism in the artificial diet were restored in response to SW. SW further decreased the concentration of short-chain fatty acids (SCFAs) in both treatments. Our results suggest that PSCs play a key role in regulating gut microbiota community and intestinal homeostasis, thereby maintaining host health.

**Key points:**

• *Swainsonine improves the intestinal bacterial diversity of plateau pikas*.

• *Swainsonine promotes the recovery of core bacterial abundances in the gut of plateau pikas*.

• *Swainsonine promotes the restoration of intestinal bacterial functions of plateau pikas*.

**Supplementary Information:**

The online version contains supplementary material available at 10.1007/s00253-021-11478-6.

## Introduction

Over the last few decades, the livestock industry of the Qinghai–Tibet Plateau has developed rapidly, which has rendered 60% of the grassland vulnerable to degradation. Overgrazing of livestock can alter plant communities and cause soil erosion, the loss of biodiversity, and the proliferation of poisonous plants, thereby damaging the stability of the grassland ecosystem (Dong et al. 2013). Grasslands dominated by poisonous plants are favored by small herbivorous mammals, which lead to population outbreaks (Smith et al. 2019). However, the outbreak mechanism of small mammal populations caused by grassland degradation and the role of toxic plants in promoting their survival and reproduction has not been well explicated.

Poisonous plants contain a variety of plant secondary compounds (PSCs), which can lead to the poisoning or death of herbivores and the protection of plants from excessive gnawing (Green et al. 2019). Previous studies mainly focused on the antagonism of PSCs between plants and animals and rarely included mutualism. However, recent studies on gut microbiota have provided a new perspective that plants and animals establish mutualism through PSCs (Ozdal et al. 2016). Kohl and Dearing (2012) observed creosote toxins in *Larrea tridentata* that can enhance the gut microbial diversity in woodrats, thereby helping them to digest poisonous diets. Tannins change the morphology of microorganisms to produce antimicrobial activity (Shi et al. 2020); therefore, it acts as a natural defense against pathogenic infections. Tannins can also serve as a natural regulator of microbial populations in different habitats, including in the human intestine (Chung et al. 1998). Flavonoids in fruits and vegetables inhibit the growth of potentially pathogenic clostridia (Klinder et al. 2016). In addition, our previous study showed that swainsonine (SW), a toxic indolizidine alkaloid found in plants, promotes resilience and maintains diverse enterotypes in small herbivorous mammals (Fan et al. 2020). Martínez-Mota et al. (2020) observed that natural diets stabilize the native microbiota in the laboratory through feeding experiments. However, there are many differences between natural and artificial diets in terms of nutrients, trace elements, and PSCs. Thus, further studies are required to explore the effect of PSCs on the gut microbiota, illustrating the mutualism mechanism between plants and animals.

Plateau pikas (*Ochotona curzoniae*) are typical small herbivorous mammals in the Qinghai–Tibet Plateau. They are diurnal, burrowing, and non-hibernating and tend to be spatially clumped, interacting with the surrounding environment through foraging and excavating activities (Arthur et al. 2008). They are also an important indicator of ecosystem health, which helps maintain the biodiversity of the grassland ecosystem (Zhao et al. 2020). However, grassland degradation caused by overgrazing can lead to the outbreak of plateau pika populations and aggravate the deterioration of the grassland ecosystem (Smith et al. 2019). Alpine meadow degradation caused by livestock has been dominated by a variety of poisonous plants such as *Oxytropis ochrocephala*, *Oxytropis kansuensis*, *Achnatherum inebrians*, and *Astragalus variabilis*. Plants of the genus *Oxytropis* are also called “locoweeds” because they contain SW, a toxic indolizidine alkaloid that causes neurological disorders in livestock (Lu et al. 2014). These plants account for approximately 33% of the damaged area caused by poisonous plants in China (Wu et al. 2014), but plateau pikas like to forage these plants as their main dietary item (Jiang and Xia 1985). In addition, short-chain fatty acids (SCFAs) are produced by the microbial fermentation of complex polysaccharides in the pika’s gut, which contribute to their nutrition and energetics (Li et al. 2018). SCFAs play a key role in various host physiological functions, such as nutrient utilization and immune regulation (Tremaroli and Bäckhed 2012). Furthermore, SCFAs might be one possible pathway for the gut microbiota to communicate with host organs and actively participate in the host’s energy homeostasis regulation (Cani and Knauf 2016; Rastelli et al. 2018). Thus, the reason for plateau pikas feeding on *Oxytropis*, the role of SW in shaping the gut bacterial community, and SCFA concentration need further elucidation.

In this study, we used plateau pika as our animal model and transferred pikas from the field to the laboratory, wherein we aimed to conduct long-term and short-term treatment experiments to investigate (i) whether SW can remodel their native gut bacteria and enhance the gut bacterial diversity, and (ii) whether SW increases the complexity of the co-occurrence network and promotes the restoration of bacterial functions. We also determined the impact of SW on SCFA concentration in the long-term and short-term treatments. This work represents a step forward in understanding the role of PSCs in regulating the structure and function of gut microbiota and in illustrating the mechanism of reciprocity between plants and small herbivorous mammals with interactions between PSCs and gut microbiota.

## Materials and methods

### Sample collection

Twenty wild plateau pikas were collected in July and November 2017 from Gangcha County, Qinghai Province, China. The captured adult pikas were placed in cages that had been disinfected with 75% alcohol. Fresh fecal samples were collected in 2 mL cryotubes and frozen in liquid nitrogen before being sent to Northwest Plateau Institute of Biology, Chinese Academy of Sciences, Xining, Qinghai, for analysis. The feces of wild pikas that were collected in July and November were defined as the JW and NW groups, respectively.

### Animal maintenance and diet treatment

After feces collection in the wild, 10 pikas captured in July and 10 in November were brought to the laboratory and housed separately in a plastic box (45 cm × 32 cm × 19 cm) with free access to the artificial diet (45 g; rabbit maintenance feed, KEAO XIELI Feed Co., Ltd. Beijing, China) and water. Individuals captured in July were housed for 20 weeks (long-term treatment), while individuals captured in November were housed for 2 weeks (short-term treatment). The feces were collected at the end of the treatments and named JC and NC groups, respectively. For four weeks, SW was extracted and added to the artificial diet for the long-term and short-term treatments. The feces were collected again at the end of the experiment and named JCS and NCS groups, respectively.

SW was extracted by the ultrasonic-chloroform extraction method from *O. ochrocephala* plants harvested near the sampling sites, and the final purity was ~ 93% (Liu et al. 2006). Then, SW was dissolved in distilled water at a concentration of 0.1 mg/mL, sprayed evenly on the quantitative feed, and dried for packaging in sealed bags. The dose of SW was determined by the daily intake of *Oxytropis* by plateau pikas under natural conditions (Jiang and Xia 1985). Therefore, 0.1 mg SW was added to each individual’s daily diet.

### Determination of fecal SCFAs

Five main SCFAs were measured using propyl chloroformate (PCF) derivatization, followed by gas chromatography-mass spectrometry (GC–MS) according to protocols described previously (Zheng et al. 2013). Each fecal sample (0.1 g) was added to 1000 µL of 0.005 M aqueous sodium hydroxide (NaOH) containing the internal standard (5 µg/mL caproic acid-d3), homogenized for 10 min, and centrifuged at 13,000 rpm and 4 °C for 20 min. The supernatant was aliquoted and transferred to a 15 mL BBI® topped cap centrifuge tube (F600888; BBI Life Sciences Corporation, Shanghai, China). Then, 100 µL of PCF, 500 µL of PrOH/Pysolution (3:2, v/v), and 300 µL of water were added to the aliquot, which was vortexed for 30 min. The derivatization reaction proceeded for 1 min under ultrasonication. After 300 μL hexane was added in the first extraction, the reaction mixture was vortexed for 1 min and then centrifuged at 2000 rpm for 5 min. Next, the supernatant layer was transferred to an autosampler vial. An additional 200 µL of hexane was used for the second extraction, and a total of 500 µL of derivatized extract was collected in the autosampler vial. Approximately 10 mg of anhydrous sodium sulfate was added to remove traces of water from hexane in an autosampler vial. The mixture was briefly vortexed prior to GC–MS analysis. An Agilent 7890A/5975C GC–MS system (MSD; Agilent Technologies, Santa Clara, CA, USA) was used to perform the analysis. Derivatives were separated using an HP-5MS capillary column (Agilent J&W Scientific, Folsom, CA, USA). One milliliter of the derivatized sample was injected in split mode (split ratio, 10:1), and the solvent delay time was set to 2.5 min. The initial oven temperature was maintained at 50 °C for 2 min; subsequently increased to 70 °C at a rate of 10 °C min^−1^, 85 °C at 3 °C min^−1^, 110 °C at 5 °C min^−1^, and 290 °C at 30 °C min^−1^; and finally held at 290 °C for 8 min. The carrier gas was helium, and the constant flow rate was 1 mL min^−1^. The front inlet, transfer line, and electron affect ion source temperatures were set at 260 °C, 290 °C, and 230 °C, respectively. The electron energy was − 70 eV, and the full scan mode (m/z 30–600) was used to collect the mass spectral data.

### DNA extraction and sequencing

According to the standard protocol, DNA was extracted from the fecal samples using QIAamp DNA Stool Mini Kit (Qiagen, Dusseldorf, Germany). A NanoDrop ND-1000 system (Thermo Fisher Scientific, Waltham, MA, USA) was used to determine the DNA concentration. Universal primers 341F (5′-CCTAYGGGRBGCASCAG-3′) and 806R (5′-GGACTACNNGGGTATCTAAT-3′) were used to amplify the V3–V4 regions of 16S rDNA. The polymerase chain reaction (PCR) products were quantified and purified using a QuantiFluor™ fluorometer (Promega Biotech, Madison, WI, USA). Negative controls included no templates for DNA extraction or PCR amplification. The PCR products were mixed in equidensity ratios. The mixture of the PCR products was purified with a Gel Extraction Kit (Qiagen). Sequencing libraries were generated using the TruSeq® DNA PCR-Free Sample Preparation Kit (Illumina, San Diego, CA, USA) following manufacturer’s instructions, with the addition of index codes. The library quality was assessed on the Qubit@ 2.0 Fluorometer (Thermo Fisher Scientific) and Bioanalyzer 2100 system (Agilent). Finally, the library was sequenced on a HiSeq2500 platform (Illumina), and 250 bp paired-end reads were generated.

### Bioinformatics and statistical analysis

The raw sequences were analyzed using the Quantitative Insights into Microbial Ecology (QIIME) Pipeline Version 1.9.1 (Caporaso et al. 2010). After the low-quality sequences and chimeras were removed, the sequences were assigned into operational taxonomic units (OTUs) using a confidence threshold of 97%. OTUs belonging to the mitochondrion or chloroplast were removed. The original data were pooled together, and all effective reads were searched against the Silva reference database (https://www.arb-silva.de). Alpha/beta diversity and abundance were determined using Mothur v1.39.1 with default settings (Schloss et al. 2009). The bacterial function was calculated by Tax4Fun (http://tax4fun.gobics.de) using the Kyoto Encyclopedia of Genes and Genomes (KEGG) database (https://www.kegg.jp).

The core bacteria were defined from different taxa. The top 10 bacteria were based on the relative abundance at the phylum level, and the top 15 bacteria were based on the relative abundance at the family level and the OTUs present in more than 80% of the samples. Statistical analyses were performed using R v4.0.2 (R Core Team, 2020) and SPSS v21.0 (IBM Corp., Armonk, NY, USA). Non-metric multidimensional scaling (NMDS) was performed using “vegan v2.5–6” (Dixon 2003) based on the Bray–Curtis distances of the OTUs and metabolism categories. Significant differences in the alpha diversity, phylum, and family relative abundances after inverse normal transformation among different groups were assessed using the Kruskal–Wallis test. The pairwise analysis of core OTU abundance, alpha diversity, and KEGG Metabolism category were assessed by the Wilcoxon rank-sum test. The linear discriminant analysis effect size (LEfSe) (http://huttenhower.sph.harvard.edu/lefse) was used to evaluate the differences in bacterial communities among groups. The core OTU abundance was first inverse normal transformed, and Spearman’s correlation values along with groupings were plotted using “pheatmap.” Spearman’s correlation of the top 30 bacteria at the family level in each group was analyzed using “psych” (Yuen et al. 2018), and the results were presented as a network using Gephi v0.9.2 (Jacomy et al. 2014). The SCFA concentrations were analyzed using one-way analysis of variance followed by Dunnett’s T3 test. Statistical significance was set at a *p* < 0.05 after the Benjamini–Hochberg false discovery rate (FDR) correction (Benjamini and Hochberg 1995).

### Accession numbers

All raw sequences in this study were submitted to the National Center for Biotechnology Information database with accession number PRJNA613933.

## Results

### Changes in the diversity and structure of gut bacteria

We identified the clustering of sequences with 97% similarity into 11,740 OTUs, and 8696 and 8160 OTUs were identified for the long-term and short-term treatments, respectively.

**Fig. 1:**
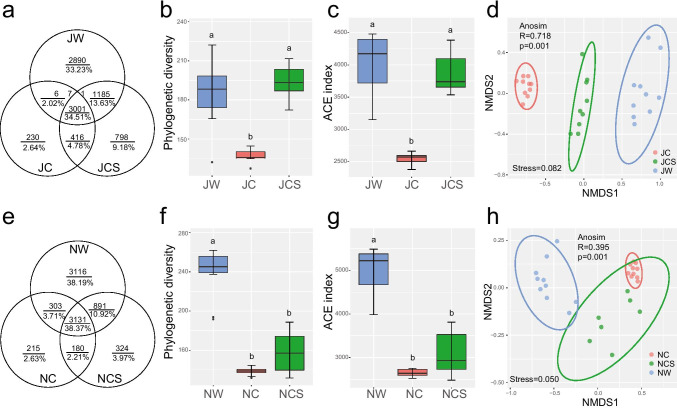
Changes in the diversity and structure of the gut bacterial community. (**a**) OTU distribution, (**b**) phylogenetic diversity, (**c**) ACE index, and (**d**) NMDS based on the Bray–Curtis distances at the OTU level in the long-term treatment. (**e**) OTU distribution, (**f**) phylogenetic diversity, (**g**) ACE index, and (**h**) NMDS based on the Bray–Curtis distances at the OTU level in the short-term treatment. All pairwise analyses of the Kruskal–Wallis test were used for post hoc multiple comparisons, and significant differences are marked by different letters (*p* < 0.01)

The Venn diagram showed that only 176 (2.02%) OTUs were shared between the JW and JC groups, while 1185 (13.63%) OTUs were shared between the JW and JCS groups in the long-term treatment (Fig. 1a). The phylogenetic diversity; abundance-based coverage estimator (ACE); and Shannon, Chao1, and observed species indices of the JW and JCS groups were significantly higher than those of the JC group (Fig. 1b, c; Supplemental Fig. S1a). The results of the NMDS analysis based on the Bray–Curtis distance showed significant differences in the bacterial communities in the long-term treatment (analysis of similarity (ANOSIM), *R* = 0.718, *p* = 0.001) (Fig. 1d). Furthermore, pairwise comparisons indicated significant differences between any two groups (JW vs JC, ANOSIM, *R* = 1.000, *p* = 0.001; JW vs JCS, ANOSIM, *R* = 0.751, *p* = 0.001; JC vs JCS, ANOSIM, *R* = 0.725, *p* = 0.001).

Only 303 (3.71%) OTUs were shared between the NW and NC groups, while 891 (10.92%) OTUs were shared between the NW and NCS groups in the short-term treatment (Fig. 1e). The alpha diversity index of the NW group was significantly higher than that of the NC and NCS groups (Fig. 1f, g; Supplemental Fig. S1b). NMDS analysis based on the Bray–Curtis distance revealed significant differences in the bacterial communities in the short-term treatment (ANOSIM, *R* = 0.395, *p* = 0.001) (Fig. 1h). Furthermore, there were significant differences between the NW and NC groups (ANOSIM, *R* = 0.480, *p* = 0.001), and between the NC and NCS groups (ANOSIM, *R* = 0.604, *p* = 0.001), while no significant difference was observed between the NW and NCS groups (ANOSIM, *R* = 0.125, *p* = 0.086). In addition, the Wilcoxon test showed that the alpha diversity in the NW group was significantly higher than that in the JW group, while only the Shannon index was significantly different between the JC and NC groups, after pikas were transferred from the wild to the laboratory (Supplemental Fig. S2).

### Composition and changes in the core bacteria

**Fig. 2:**
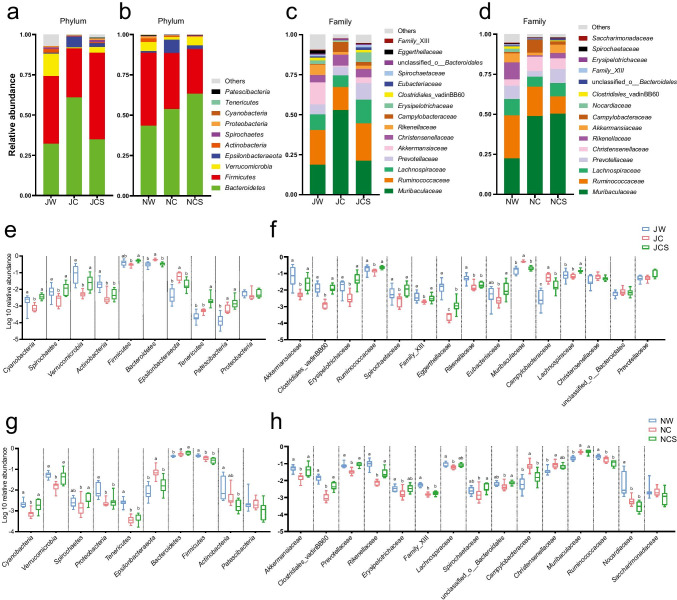
Composition and changes of the gut bacteria. The composition and changes of core bacteria at the (**a**, **e**) phylum and (**c**, **f**) family level in the long-term treatment. The composition and changes of core bacteria at the (**b**, **g**) phylum and (**d**, **h**) family level in the short-term treatment. All pairwise analyses of the Kruskal–Wallis test were used for post hoc multiple comparisons, and significant differences are marked by different letters (FDR *p* < 0.05)

We defined the top 10 bacteria at the phylum level and top 15 at the family level as core bacteria for analyzing species composition and changes among groups (Fig. 2a–d). In the long-term treatment, the relative abundances of the phyla *Cyanobacteria*, *Spirochaetes*, and *Verrucomicrobia* in the JW and JCS groups were significantly higher than those of the JC group, while the relative abundances of *Bacteroidetes* and *Epsilonbacteraeota* were significantly lower than those of the JC group (Fig. 2e; Supplemental Table S1). At the family level, the relative abundances of *Akkermansiaceae*, *Clostridiales_*vadinBB60, *Erysipelotrichaceae*, *Ruminococcaceae*, *Spirochaetaceae*, and *Family_* XIII were significantly higher, while that of *Muribaculaceae* was significantly lower in the JW and JCS groups than in the JC group (Fig. 2f; Supplemental Table S1). In the short-term treatment, the relative abundances of the phyla *Cyanobacteria* and *Verrucomicrobia* in the NW and NCS groups were significantly higher than those of the NC group, while that of *Epsilonbacteraeota* was significantly lower than that of the NC group (Fig. 2g; Supplemental Table S2). At the family level, *Akkermansiaceae*, *Clostridiales*_vadinBB60, *Prevotellaceae*, and *Rikenellaceae* were significantly more abundant, while *Campylobacteraceae* was significantly less abundant in the NW and NCS groups compared to the NC group (Fig. 2h; Supplemental Table S2).

**Fig. 3:**
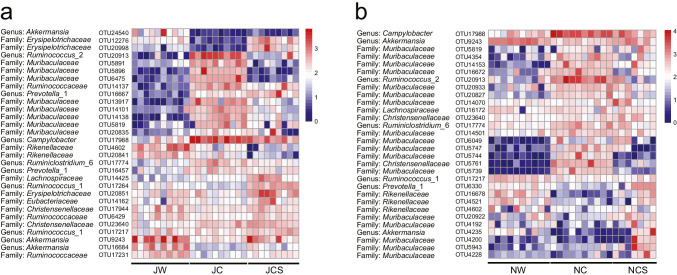
Taxonomic alterations in the gut bacteria. Changes in the log-abundance of core OTUs in the (**a**) long-term and (**b**) short-term treatments. Core OTUs are shown for the lower taxonomic unit

LEfSe analysis was used to determine the taxa that most likely explained the differences between pikas from different groups at the phylum and family level. Pikas in the JC/NC group were enriched in fewer bacteria compared with the JW/NW and JCS/NCS groups (Supplemental Fig. S3 and Fig. S4). We identified core OTUs that were present across 80% of fecal samples in each treatment and detected the top 30 core OTUs in the two treatments. In the long-term treatment, the most abundant was *Campylobacter* (OTU17988), followed by *Akkermansia* (OTU9243), *Ruminococcus_2* (OTU20913), and *Erysipelotrichaceae* (OTU20851). Wilcoxon’s test showed that diet treatments significantly affected core OTU abundances in the laboratory (Table 1). That is, the abundances of 26 core OTUs significantly differed between the JW and JC groups, of which 10 OTUs were reduced and 16 were increased in pikas from the wild compared to the laboratory. In contrast, there were 19 core OTUs that differed between the JW and JCS groups (Fig. 3a). Moreover, pikas fed the artificial diet in the laboratory showed a drastic reduction in core OTUs of the genus *Akkermansia* (OTU24540, 9243, 16,684) and the family *Ruminococcaceae* (OTU6429, 17,231). In the short-term treatment, the most abundant OTU was *Campylobacter* (OTU17988), followed by *Ruminococcus*_2 (OTU20913), *Akkermansia* (OTU9243), and *Christensenellaceae* (OTU5761). Wilcoxon’s test revealed that the abundances of 21 core OTUs significantly differed between the NW and NC groups (Table 2), of which six OTUs were reduced and 15 were increased in pikas that were transferred from the wild to the laboratory. By comparison, the abundance of only eight core OTUs differed between the NW and NCS groups (Fig. 3b). Additionally, pikas fed the artificial diet in the laboratory demonstrated a drastic reduction in core OTUs of the genera *Akkermansia* (OTU9243, 4235) and *Ruminococcus*_1 (OTU17217) and the family *Rikenellaceae* (OTU16678, 4521).

**Table 1:** Results of the Wilcoxon rank-sum test comparing the abundance of core OTU between plateau pikas in the JW and JC groups, and JW and JCS groups

Taxonomic classification	Core OTU	JW vs JC		JW vs JCS	
		Z	FDR *p*	Z	FDR *p*
Genus: *Akkermansia*	OTU24540	**3.792**	** < 0.001**	**3.599**	** < 0.001**
Family: *Erysipelotrichaceae*	OTU12276	**3.788**	** < 0.001**	** − 2.694**	** < 0.01**
Family: *Erysipelotrichaceae*	OTU20998	**3.077**	** < 0.01**	** − 3.184**	** < 0.01**
Genus: *Ruminococcus*_2	OTU20913	** − 3.785**	** < 0.001**	** − 2.536**	** < 0.05**
Family: *Muribaculaceae*	OTU5891	** − 3.780**	** < 0.001**	0.816	0.488
Family: *Muribaculaceae*	OTU5896	** − 3.798**	** < 0.001**	0.125	0.920
Family: *Muribaculaceae*	OTU6475	** − 3.780**	** < 0.001**	0.368	0.758
Family: *Ruminococcaceae*	OTU14137	** − 3.782**	** < 0.001**	** − 3.677**	** < 0.001**
Genus: *Prevotella*_1	OTU16667	** − 3.784**	** < 0.001**	** − 3.679**	** < 0.001**
Family: *Muribaculaceae*	OTU13917	** − 3.637**	** < 0.001**	** − 3.520**	** < 0.001**
Family: *Muribaculaceae*	OTU14101	** − 3.176**	** < 0.01**	** − 2.369**	** < 0.05**
Family: *Muribaculaceae*	OTU14138	** − 3.788**	** < 0.001**	** − 3.193**	** < 0.01**
Family: *Muribaculaceae*	OTU5819	** − 2.874**	** < 0.01**	− 1.635	0.138
Family: *Muribaculaceae*	OTU20835	** − 3.707**	** < 0.001**	− 0.899	0.462
Genus: *Campylobacter*	OTU17988	** − 3.780**	** < 0.001**	** − 3.184**	** < 0.01**
Family: *Rikenellaceae*	OTU4602	**3.477**	** < 0.001**	**3.512**	** < 0.001**
Family: *Rikenellaceae*	OTU20841	**2.797**	** < 0.01**	**3.674**	** < 0.001**
Genus: *Ruminiclostridium*_6	OTU17774	** − 3.628**	** < 0.001**	** − 2.613**	** < 0.05**
Genus: *Prevotella*_1	OTU16457	** − 2.646**	** < 0.05**	− 1.715	0.121
Family: *Lachnospiraceae*	OTU14425	** − 3.176**	** < 0.01**	** − 3.676**	** < 0.001**
Genus: *Ruminococcus*_1	OTU17264	**2.873**	** < 0.01**	** − 3.511**	** < 0.001**
Family: *Erysipelotrichaceae*	OTU20851	0.794	0.484	** − 3.103**	** < 0.01**
Family: *Eubacteriaceae*	OTU14162	1.058	0.371	− 1.143	0.334
Family: *Christensenellaceae*	OTU17944	0.832	0.484	− 0.245	0.871
Family: *Ruminococcaceae*	OTU6429	**2.609**	** < 0.05**	0.449	0.708
Family: *Christensenellaceae*	OTU23640	** − 2.873**	** < 0.01**	** − 3.429**	** < 0.001**
Genus: *Ruminococcus*_1	OTU17217	− 0.076	0.971	− 2.041	0.056
Genus: *Akkermansia*	OTU9243	**3.553**	** < 0.001**	1.633	0.138
Genus: *Akkermansia*	OTU16684	**3.553**	** < 0.001**	**2.449**	** < 0.05**
Family: *Ruminococcaceae*	OTU17231	**3.780**	** < 0.001**	**2.941**	** < 0.01**

Significant effects are in bold

**Table 2:** Results of the Wilcoxon rank-sum test comparing the abundance of core OTU between plateau pikas in the NW and NC groups, and NW and NCS groups

Taxonomic classification	Core OTU	NW vs NC		NW vs NCS	
		Z	FDR *p*	Z	FDR *p*
Genus: *Campylobacter*	OTU17988	**3.780**	** < 0.001**	2.049	0.081
Genus: *Akkermansia*	OTU9243	** − 3.628**	** < 0.001**	− 1.854	0.127
Family: *Muribaculaceae*	OTU5819	**2.609**	** < 0.05**	2.049	0.081
Family: *Muribaculaceae*	OTU4354	**2.949**	** < 0.01**	1.562	0.216
Family: *Muribaculaceae*	OTU14153	**3.784**	** < 0.001**	**3.422**	** < 0.001**
Family: *Muribaculaceae*	OTU16672	**3.100**	** < 0.01**	0.636	0.656
Genus: *Ruminococcus*_2	OTU20913	**3.780**	** < 0.001**	0.390	0.838
Family: *Muribaculaceae*	OTU20933	**2.419**	** < 0.05**	1.171	0.377
Family: *Muribaculaceae*	OTU20827	**3.780**	** < 0.001**	**2.537**	** < 0.05**
Family: *Muribaculaceae*	OTU14070	− 0.151	0.927	0.390	0.838
Family: *Lachnospiraceae*	OTU16172	0.529	0.757	0.000	1.000
Family: *Christensenellaceae*	OTU23640	1.399	0.248	**2.733**	** < 0.05**
Genus: *Ruminiclostridium*_6	OTU17774	**3.175**	** < 0.01**	0.683	0.656
Family: *Muribaculaceae*	OTU14501	**3.100**	** < 0.01**	2.148	0.066
Family: *Muribaculaceae*	OTU6049	**3.798**	** < 0.001**	**2.424**	** < 0.05**
Family: *Muribaculaceae*	OTU5747	**3.784**	** < 0.001**	0.198	0.918
Family: *Muribaculaceae*	OTU5744	**3.797**	** < 0.001**	1.389	0.282
Family: *Christensenellaceae*	OTU5761	**3.797**	** < 0.001**	**3.246**	** < 0.001**
Family: *Muribaculaceae*	OTU5739	**3.801**	** < 0.001**	**3.450**	** < 0.001**
Genus: *Ruminococcus*_1	OTU17217	** − 3.402**	** < 0.001**	** − 2.635**	** < 0.05**
Genus: *Prevotella*_1	OTU6330	** − 3.402**	** < 0.001**	0.976	0.485
Family: *Rikenellaceae*	OTU16678	** − 3.781**	** < 0.001**	− 1.073	0.430
Family: *Rikenellaceae*	OTU4521	** − 3.441**	** < 0.001**	− 1.561	0.216
Family: *Rikenellaceae*	OTU4602	0.302	0.856	0.293	0.856
Family: *Muribaculaceae*	OTU20922	− 0.303	0.856	1.465	0.248
Family: *Muribaculaceae*	OTU4192	− 0.416	0.805	0.878	0.532
Genus: *Akkermansia*	OTU4235	** − 3.371**	** < 0.001**	− 0.293	0.856
Family: *Muribaculaceae*	OTU4200	− 0.911	0.513	1.222	0.329
Family: *Muribaculaceae*	OTU5943	1.816	0.132	**2.685**	** < 0.05**
Family: *Muribaculaceae*	OTU4228	− 1.550	0.211	1.464	0.248

Significant effects are in bold

### Co-occurrence network of the core bacteria

**Fig. 4:**
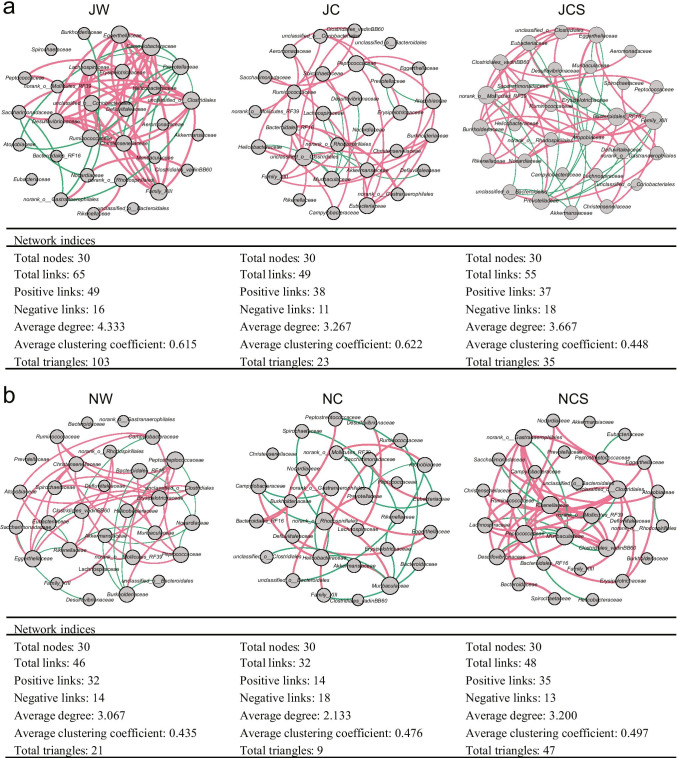
Co-occurrence networks of core bacteria. Co-occurrence networks and topological indices of the top 30 bacteria at the family level in the (**a**) long-term and (**b**) short-term treatments. Spearman’s correlation greater than 0.5 or lower than − 0.5 with FDR *p* < 0.05 are illustrated. Line color reflects direction (red: positive; green: negative). Node size is proportional to the number of connections (i.e., degree)

The co-occurrence network based on the top 30 bacteria at the family level reveals the relationship between gut bacteria in pikas (Fig. 4). We observed that the complexity of the network structure was distinctly different between groups. In the long-term treatment, the JW group had 65 links, followed by the JCS (55 links) and JC (49 links) groups. Notably, the positive correlation between *Eggerthellaceae* and *Erysipelotrichaceae* and between *Christensenellaceae* and *Family*_XIII, as well as the negative correlation between *Eggerthellaceae* and *Prevotellaceae*, *Family*_XIII and *Prevotellaceae*, and *Defluviitaleaceae* and *Prevotellaceae*, which had disappeared in the JC group, reappeared in the JCS group after SW was added to the artificial diet (Fig. 4a). In the short-term treatment, the NCS group had 48 links, and the NW group had 46 links, while the NC group had 32 links. After SW was added to the artificial diet, the positive correlation between *Clostridiales*_vadinBB60 and *Rikenellaceae*, unclassified_o__*Clostridiales* and *Ruminococcaceae*, unclassified_o__*Clostridiales* and *Erysipelotrichaceae*, and the negative correlation between *Muribaculaceae* and *Rikenellaceae*, which had disappeared in the NC group, reappeared in the NCS group (Fig. 4b).

### The correlation between core bacteria and SCFAs, and between KEGG and SCFAs

NMDS analysis based on the Bray–Curtis distance showed significant differences in metabolism categories (level 3) in both the long-term and short-term treatments (long-term, ANOSIM, *R* = 0.242, *p* = 0.001; short-term, ANOSIM, *R* = 0.128, *p* = 0.024) (Supplemental Fig. S5). The Wilcoxon test showed that SW also affects the bacterial functions, irrespective of the duration of the treatment in the laboratory. In the long-term treatment, the relative abundance of sequences related to ko00250, ko00680, ko00770, and ko00860 was significantly different between the JW and JC groups but not between the JW and JCS groups (Supplemental Table S3). In the short-term treatment, the relative abundance of sequences associated with ko00250, ko00500, ko00040, ko00190, ko00511, ko00790, and ko00450 had significant differences between the NW and NC groups, while no significant differences were observed in these metabolism categories between the NW and NCS groups (Supplemental Table S4).

**Fig. 5:**
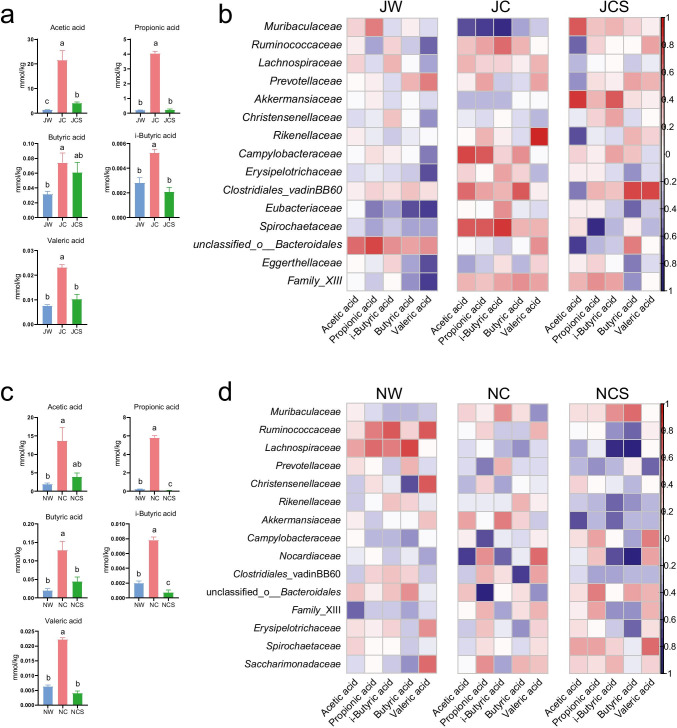
SCFA concentration and its relationship with core bacteria. Changes in the (**a**) SCFA concentration and Spearman’s correlation between (**b**) SCFAs and core bacteria at the family level in the long-term treatment. Changes in the (**c**) SCFA concentration and Spearman’s correlation between (**d**) SCFAs and core bacteria at the family level in the short-term treatment. The differences in SCFA concentration were calculated by one-way analysis of variance (ANOVA) followed by Dunnett’s T3 test. Significant differences are marked by different letters (*p* < 0.05)

The concentrations of acetic acid, propionic acid, *i*-butyric acid, and valeric acid in the JC group were significantly higher than those of the other two groups in the long-term treatment (Fig. 5a). Compared to the NW and NCS groups, the NC group had significantly higher concentrations of propionic acid, butyric acid, *i*-butyric acid, and valeric acid in the short-term treatment (Fig. 5c). Spearman’s correlation between SCFAs and core bacteria, and SCFAs and metabolism categories were also calculated in both treatments (Fig. 5b, d; Supplemental Fig. S6).

## Discussion

We showed that both long-term and short-term treatments significantly reduced the alpha diversity of plateau pikas’ gut bacteria in the laboratory. The beta diversity of gut bacteria in pikas was also significantly influenced by the shift from the field to the laboratory. These results are consistent with previous studies of captive giant pandas (Guo et al. 2019), deer mice (Schmidt et al. 2019), woodrats (Kohl et al. 2014), Tibetan wild asses (Gao et al. 2019), lizards (Kohl et al. 2017), and capercaillies (Wienemann et al. 2011). This phenomenon could be due to three reasons. First, the drastic difference in the environment would be an important factor. Evidence has shown that the composition of gut microbiota is constrained by neutral dispersion limitations (Linnenbrink et al. 2013; Burns et al. 2016). This implies that the chances of microbes successfully colonizing the environment are equal; the more the host is exposed to microbial species, the more likely the microbial species would be to remain in the host’s gut microbial community. Therefore, continued exposure to various microbial communities is necessary to maintain the microbial species and gut microbial diversity. However, laboratory conditions cause animals to live in a limited space, losing contact with the complex external environment and blocking the horizontal transmission of gut microbiota between animals (Kohl et al. 2014). Second, the artificial diet in the laboratory affects animals’ gut microbiota. A natural diet can cause differences in animal gut microbiota through the level of nutrition (Liu et al. 2018), fiber concentration (Pu et al. 2020), trace elements (Kanhere et al. 2018), and PSCs (Zhang et al. 2020). Meanwhile, the diversity of animal gut microbiota may be positively related to diet richness (Delport et al. 2016). Compared with the wild plant-based diet, the artificial diet provided in the laboratory has a significantly higher content of fat and protein but lacks a variety of trace elements. These high-fat and high-protein diets are also important reasons for the decline in microbial diversity (Zhang and Yang 2016). Third, the interruption of individual social relationships may be an important reason for the decrease in gut microbial diversity (Nelson et al. 2013). For instance, chimpanzees acquire most of their gut microbiomes through social interactions (Moeller et al. 2016). In addition, social relationships can shape the gut microbiota through the spread of shared environments (Lax et al. 2014).

The relative abundances of the core bacteria *Akkermansiaceae*, *Erysipelotrichaceae*, *Ruminococcaceae*, and *Rikenellaceae* decreased in both treatments. The trend of the relative abundance of *Ruminococcaceae* is consistent with that in a previous study of captive deer mice (Schmidt et al. 2019). Meanwhile, a study on lizards also showed that captivity decreased the relative abundances of *Ruminococcus* and *Rikenella* (Kohl et al. 2017). The members of *Ruminococcaceae* are the most widely studied bacteria in the fiber degradation process and contain numerous cellulolytic species (Koike and Kobayashi 2001). *Rikenellaceae* is a family of hydrogen-producing bacteria that can protect cells from oxidative stress injuries (Chen et al. 2011), and the abundance of *Rikenellaceae* can be increased with lactulose intervention in mice (Zhai et al. 2018). The changes in the abundance of these bacteria may be influenced by the cellulose and fructose content in the diet. In addition, it has been demonstrated that the abundance of *Akkermansia muciniphila* in *Akkermansiaceae* is negatively correlated with obesity and metabolic disorders in mice (Everard et al. 2013) and that *Erysipelotrichaceae* is related to host lipid metabolism (Kaakoush 2015). While the relative abundance of *Muribaculaceae* increased in both treatments (Fig. 2f, h), a previous study on deer mice also showed that the relative abundance of *Muribaculaceae* (previously named S24-7) has the same changing trend (Schmidt et al. 2019). Genome analysis showed that *Muribaculaceae* is versatile with respect to its complex carbohydrate degradation (Lagkouvardos et al. 2019). Most of these bacteria are involved in food digestion and nutrient metabolism. Thus, the changes in their relative abundances in the laboratory are probably caused by the induced nutrients in the diet (Yang et al. 2020). These results indicate that the influences of high-fat and high-protein diets on mammals and humans are very similar and could also imply that the humanization of artificial feeding causes animal gut microbiota to converge.

After SW was added to the artificial diet, no significant difference was observed in the alpha diversity between the JW and JCS groups in the long-term treatment, and no significant difference was found in the beta diversity between the NW and NCS groups in the short-term treatment. These results indicate that SW can increase the gut bacterial diversity and promote the restoration of the gut community structure. Research on PSCs such as creosote resin and phenolic compounds have also shown the ability to enhance gut microbial diversity (Kohl and Dearing 2012; Dominguez-Avila et al. 2020). Kohl and Dearing (2012) demonstrated that creosote resin increases the relative abundance of dominant species of the gut microbiota in herbivores. Martínez-Mota et al. (2020) found that a natural diet promotes the retention of native gut microbiota in captive rodents. The proportion of common OTUs shared by JW and JC, and by NW and NC increased after SW was added to the artificial diet under laboratory conditions. Our results also illustrate that PSCs may promote the retention of the native gut microbiota and maintain intestinal homeostasis of the gut ecosystem in small herbivorous mammals.

PSCs can also bring the relative abundance of core bacteria closer to that in the original wild state. In a previous study, adding ellagitannins (phenolics) to the human diet considerably increased the abundance of *Akkermansiaceae* (*A. muciniphila*) (Li et al. 2015). Feeding female pigs with a diet containing proanthocyanidins (phenolics) enhanced the abundance of *Ruminococcaceae* in the feces (Choy et al. 2014). These results demonstrate that PSCs have an inhibitory effect on the abundance of microbes induced by high-fat and high-protein diets, thereby promoting the restoration of the gut bacterial community to the original wild state. However, some findings are contrary to our results. For instance, a high-fat diet supplemented with quercetin (a flavonol) inhibited the growth of *Erysipelotrichaceae* in rats (Etxeberria et al. 2015). Compared with the standard pellet diet, a lichen diet rich in various PSCs reduced the relative abundance of *Rikenellaceae* in reindeer (Salgado-Flores et al. 2016). This phenomenon could be explained by the fact that various PSCs in nature have different mechanisms in animals, resulting in diverse responses of bacteria. The relative abundance of *Campylobacter* was observed to be significantly higher in captive Tibetan wild ass (Gao et al. 2019). Recent studies have shown that there may be a link between gut microbiota and chronic diseases (Roche-Lima et al. 2018). Thus, the disruption in intestinal homeostasis may cause the overgrowth of pathogenic bacteria (Pagliari et al. 2015). We observed that the relative abundance of intestinal pathogenic bacteria was reduced after SW was added to the artificial diet, which can improve the immunity of pikas, thereby resisting diseases and microbial infections and improving population survival.

In a controlled diet experiment of voles, the group that was fed on a diet with more tannins developed a more complex microbial network (Li et al. 2019). To obtain sufficient energy from the wild diet and artificial diet with SW, pikas may need to harbor a more diverse gut bacterial community and develop a complex network to reduce the toxicity of SW in the wild and artificial diets containing SW. The SW concentration in toxic plants in July is higher than that in November (Achata Böttger et al. 2012), which may explain the higher average degree of complexity of the JW group than of the NW group. This pattern has also been confirmed in our controlled diet experiment. Meanwhile, complex microbial networks with increased connectivity are more resistant to external perturbations than simple networks are (Santolini and Barabasi 2018). This may explain why the average degree of complexity in the long-term treatment was greater than that in the short-term treatment. The average degree of complexity of the JCS group was lower than that in the JW group, but it was higher in the NCS group than in the NW group. One possible reason is the inconsistent housing duration for artificial diets in the long-term and short-term treatments, which may lead to different recovery levels after the addition of SW. Notably, the relationships between core bacteria in the network, which disappeared in the artificial diet, reappeared after the addition of SW. For example, the negative correlation between *Eggerthellaceae* and *Prevotellaceae* in the long-term treatment: several species from *Eggerthellaceae* can metabolize PSCs (Beltran et al. 2018; Bode et al. 2013), while *Prevotellaceae*, a family of beneficial microbes in digestive tract, is closely related to the decomposition of plant-derived dietary components (Ley 2016). Thus, this oppositional relationship can be seen in animals where, upon consumption of poisonous plants, they increase their ability to metabolize PSCs at the expense of reducing digestion efficiency to avoid self-poisoning.

Several metabolism categories were changed when pikas were transferred from the wild to the laboratory. These changes were most likely associated with the nutritional properties of the artificial diets. Specifically, the artificial diet was rich in carbohydrates and supplemented with 10 amino acids, while it lacked SW commonly found in the natural diet of wild pikas. The bacterial functions were restored to varying degrees after SW was added to the artificial diet in both treatments. These results indicate that PSCs could also affect gut microbiota function under laboratory conditions. The concentrations of five SCFAs we measured in the experiment were significantly higher in the artificial diet compared with the wild diet in both treatments. The artificial diet with high fat and high protein may disrupt the homeostasis of gut microbiota, increasing SCFA production and lowering the pH of the intestine, which may lead to acidosis (Bilal et al. 2016) and intestinal inflammation (Chang et al. 2019). However, SCFA concentrations were decreased after SW was added to the artificial diet in the two treatments. These results imply that PSCs may reduce the production of SCFAs and prevent acidosis in the host by regulating the abundance of gut microbiota and maintaining the microbial homeostasis of gut ecosystem.

Thus, we revealed that SW could remodel the structure and function of the gut bacteria of plateau pikas in long-term and short-term treatments. Upon transferring pikas from the wild to the laboratory, the alpha diversity was significantly reduced and beta diversity significantly differed, and the relative abundance of core bacteria was significantly altered as well. After SW was added to the artificial diet, no significant difference was observed in the alpha diversity in the long-term treatment or beta diversity in the short-term treatment compared with that in the wild, and the relative abundance of core bacteria was restored in both treatments. The interactions in the co-occurrence network that disappeared in the artificial diet reappeared to enhance the complexity of the network structure, and the abundance of pathogenic bacteria that were enriched in the artificial diet was reduced in both treatments to benefit host health. SW further recovered several metabolism categories and decreased SCFA concentration in both treatments; this decrease might prevent acidosis in the host. Therefore, we highlight the importance of PSCs in maintaining the microbial homeostasis of the gut ecosystem. This study deepens our understanding of the reciprocity mechanism between plants and small herbivorous mammals in terms of interaction between PSCs and gut microbiota.

## Supplementary Information

Below is the link to the electronic supplementary material.

Supplementary file1 (PDF 1.17 MB)

## Data Availability

Data and material described in this study are available from the authors upon reasonable request and availability.
