# Supplementary material for: The plant secondary compound swainsonine reshapes gut microbiota in plateau pikas (Ochotona curzoniae)
Source: Appl Microbiol Biotechnol. 2021 Aug 17;105(16-17):6419–33. doi: 10.1007/s00253-021-11478-6 (PMC8403131; doi:10.1007/s00253-021-11478-6)
Supplement: Supplementary file 1 — Supplementary file1 (PDF 1.17 MB) [file 253_2021_11478_MOESM1_ESM.pdf]

Supplementary material to **Applied Microbiology and Biotechnology**

The plant secondary compound swainsonine reshapes gut ~~bacteria~~ microbiota in plateau pikas (*Ochotona curzoniae*)

Shien Ren [1, 2, 3 †], Chao Fan [1, 2, 3 †], Liangzhi Zhang [1, 4 †], Xianjiang Tang [1, 2, 3], Haibo Fu [1, 2, 3], Chuanfa Liu [1, 2], Shangang Jia [5, *], Yanming Zhang [1, 2, *]

[1] Key Laboratory of Adaptation and Evolution of Plateau Biota, Northwest Institute of Plateau Biology, Chinese Academy of Sciences, Xining 810008, China

[2] Qinghai Provincial Key Laboratory of Animal Ecological Genomics, Xining 810008, China

[3] University of Chinese Academy of Sciences, Beijing 100049, China

[4] Key Laboratory of Restoration Ecology of Cold Area in Qinghai Province, Northwest Institute of Plateau Biology, Chinese Academy of Sciences, Xining 810008, China

[5] College of Grassland Science and Technology, China Agricultural University, Beijing 100193, China

† These authors are equally contributed to this work.

* Corresponding authors: shangang.jia@cau.edu.cn (S.J.); zhangym@nwipb.cas.cn (Y.Z.)

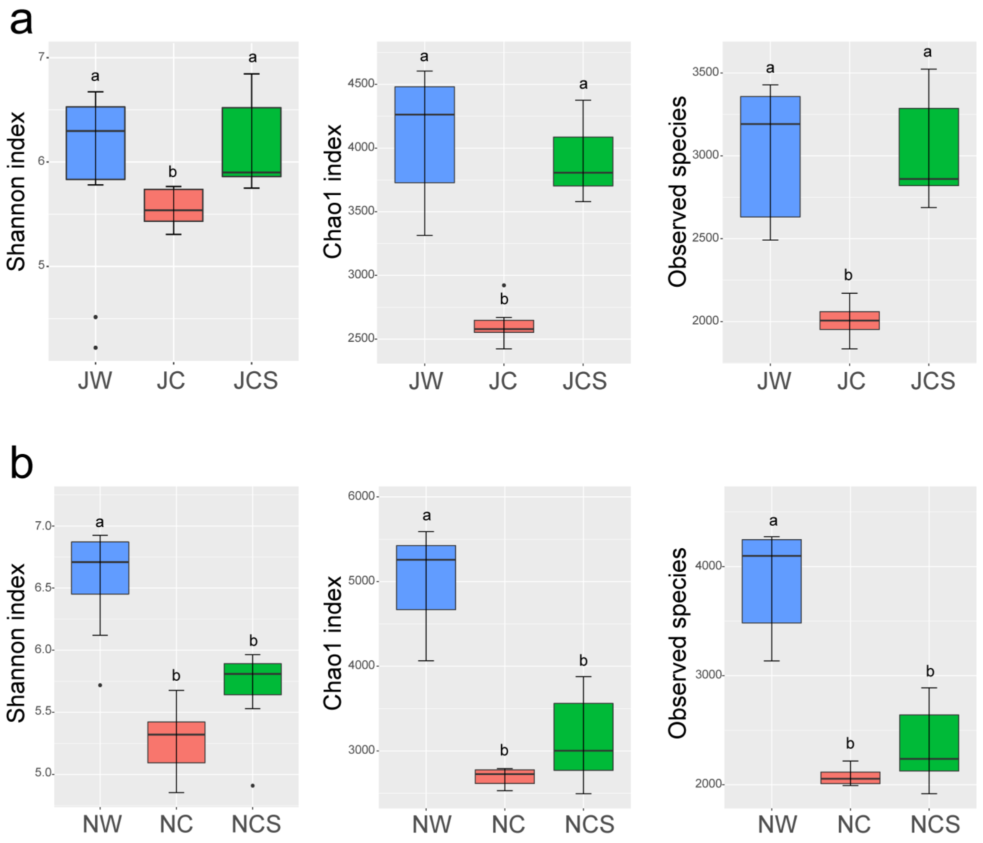

Fig. S1 The changes in the gut bacterial diversity. Shannon index, Chao1 index, and Observed species in the (**a**) long-term and (**b**) short-term treatments. All pairwise analyses of the Kruskal–Wallis test was used for post hoc multiple comparisons, and significant differences are marked by different letters ($p < 0.05$).

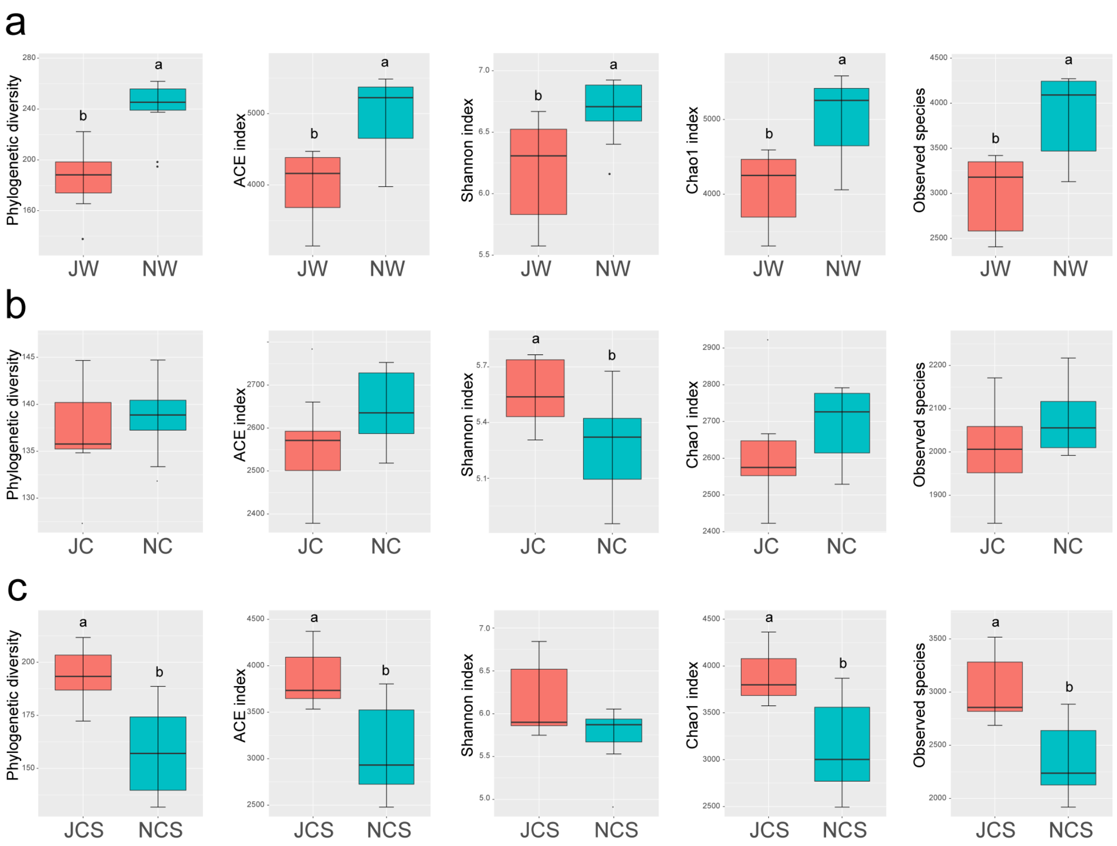

Fig. S2 The differences in the gut bacterial diversity. Phylogenetic diversity, ACE index, Shannon index, Chao1 index and Observed species between the (**a**) JW and NW groups, (**b**) JC and NC groups, and (**c**) JCS and NCS groups. The differences were calculated by the Wilcoxon rank-sum test and significant differences are marked by different letters ($p < 0.05$).

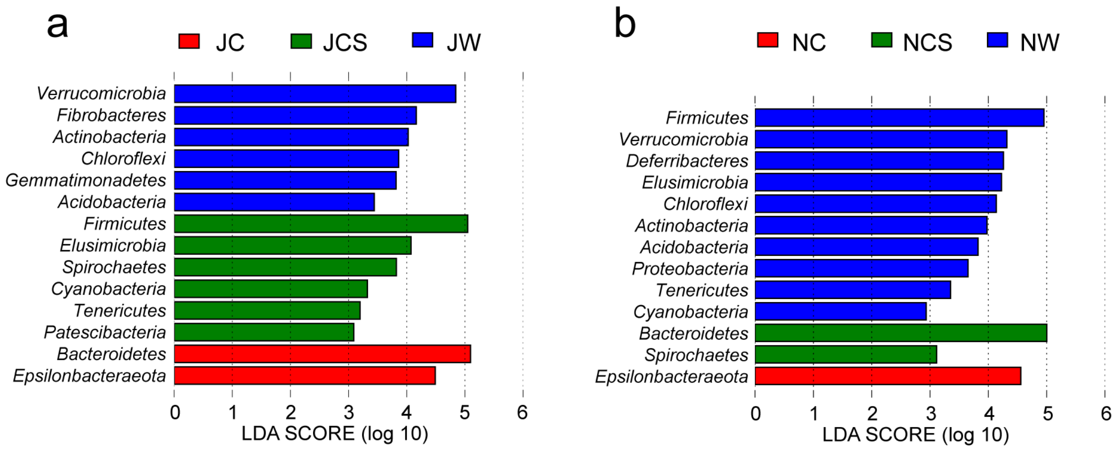

Fig. S3 Taxonomic alterations of the gut bacteria. All-against-all in the (**a**) long-term and (**b**) short-term treatments by the LEfSe method (LDA > 2, $p < 0.05$) at the phylum level.

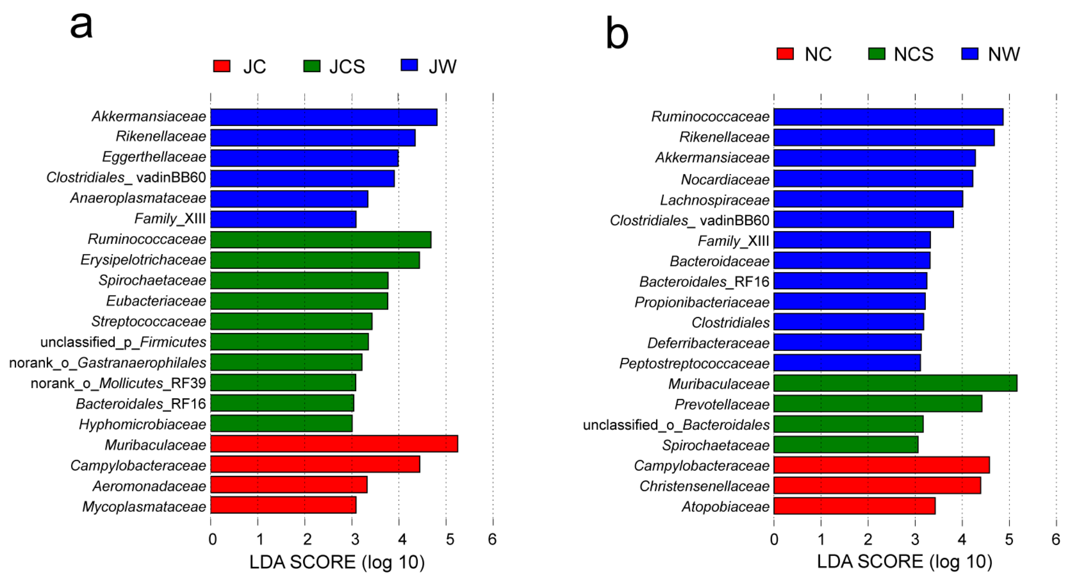

Fig. S4 Taxonomic alterations of the gut bacteria. All-against-all in the (**a**) long-term and (**b**) short-term treatments by the LEfSe method (LDA > 3, $p < 0.05$) at the family level.

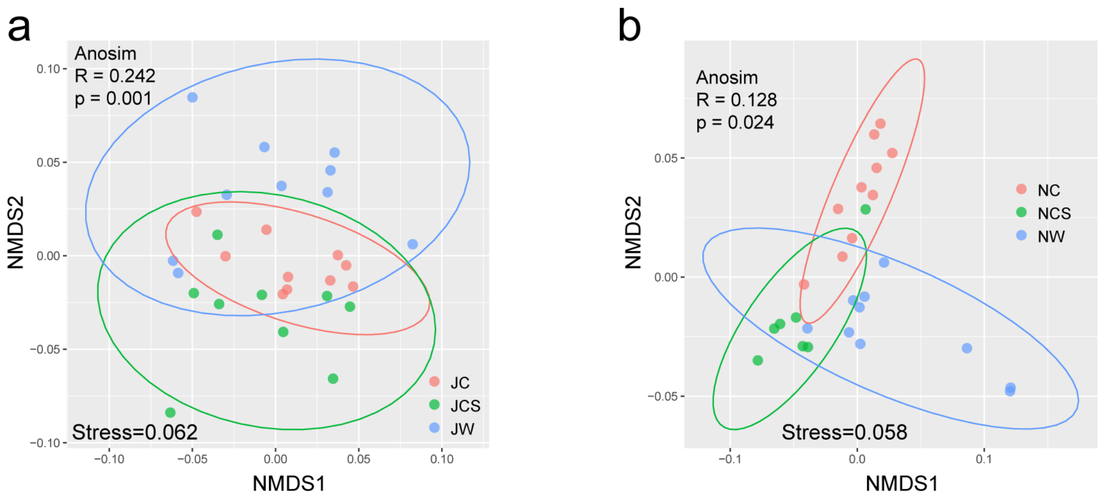

Fig. S5 The beta diversity of the KEGG categories. Non-metric multidimensional scaling based on Bray–Curtis distance of the KEGG Metabolism category (level 3) in the (**a**) long-term and (**b**) short-term treatments.

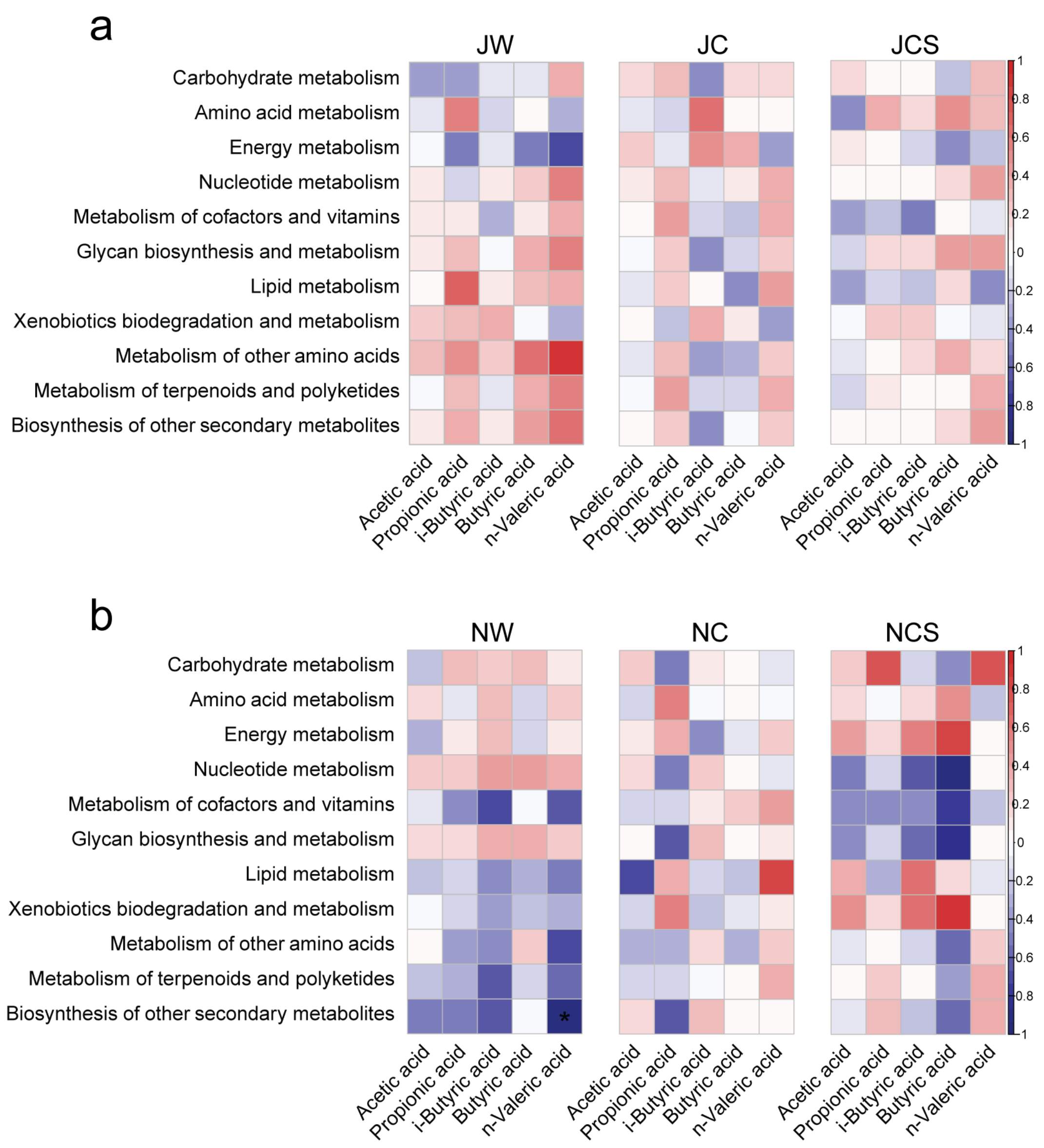

Fig. S6 The correlation between SCFAs and KEGG categories. Spearman's correlation between SCFAs concentration and KEGG Metabolism categories (level2) in the (**a**) long-term and (**b**) short-term treatments.

Table S1 Differences in the relative abundance of core bacteria among groups in the long-term treatment.

**Taxa**	**Core bacteria**	**Relative abundance (mean)**			**Kruskal-Wallis test**	
		**JW**	**JC**	**JCS**	**Test-statistic**	**FDR *p***
Phylum	*Cyanobacteria*	0.002	0.001	0.004	16.804	< 0.001
	Spirochaetes	0.009	0.003	0.014	12.096	0.002
	Verrucomicrobia	0.139	0.006	0.036	18.985	< 0.001
	Actinobacteria	0.024	0.004	0.006	17.211	< 0.001
	Firmicutes	0.419	0.305	0.538	12.865	0.002
	Bacteroidetes	0.321	0.609	0.348	19.186	< 0.001
	Epsilonbacteraeota	0.006	0.066	0.023	19.699	< 0.001
	Tenericutes	0.000	0.001	0.003	21.575	< 0.001
	Patescibacteria	0.000	0.001	0.002	18.591	< 0.001
	Proteobacteria	0.006	0.005	0.007	4.078	0.130
Family	*Akkermansiaceae*	0.139	0.006	0.036	17.642	< 0.001
	*Clostridiales*_vadinBB60	0.018	0.001	0.014	19.012	< 0.001
	Erysipelotrichaceae	0.023	0.004	0.061	17.962	< 0.001
	Ruminococcaceae	0.217	0.144	0.233	10.620	0.007
	Spirochaetaceae	0.009	0.003	0.014	10.993	0.006
	*Family*_XIII	0.005	0.002	0.003	8.064	0.024
	Eggerthellaceae	0.020	0.000	0.002	22.902	< 0.001
	Rikenellaceae	0.063	0.016	0.022	16.438	< 0.001
	Eubacteriaceae	0.008	0.003	0.016	7.902	0.024
	Muribaculaceae	0.187	0.528	0.211	19.571	< 0.001
	Campylobacteraceae	0.005	0.064	0.022	20.701	< 0.001
	Lachnospiraceae	0.097	0.072	0.148	12.690	0.003
	Christensenellaceae	0.046	0.069	0.051	4.636	0.105
	unclassified_o__*Bacteroidales*	0.007	0.009	0.008	1.543	0.462
	Prevotellaceae	0.062	0.055	0.104	5.535	0.073

Table S2 Differences in the relative abundance of core bacteria among groups in the short-term treatment.

Taxa	Core bacteria	Relative abundance (mean)			Kruskal-Wallis test	
		NW	NC	NCS	Test-statistic	FDR *p*
Phylum	*Cyanobacteria*	0.002	0.001	0.002	13.361	0.001
	Verrucomicrobia	0.055	0.016	0.053	14.728	0.001
	Spirochaetes	0.003	0.002	0.004	6.562	0.042
	Proteobacteria	0.011	0.002	0.004	14.776	0.001
	Tenericutes	0.003	0.000	0.001	18.883	< 0.001
	Epsilonbacteraeota	0.010	0.083	0.022	17.701	< 0.001
	Bacteroidetes	0.434	0.537	0.632	13.357	0.001
	Firmicutes	0.453	0.347	0.276	15.644	<0.001
	Actinobacteria	0.023	0.007	0.002	11.919	0.005
	Patescibacteria	0.004	0.002	0.002	4.424	0.110
Family	*Akkermansiaceae*	0.055	0.016	0.053	14.309	0.002
	*Clostridiales*_vadinBB60	0.015	0.001	0.005	21.395	< 0.001
	Prevotellaceae	0.083	0.035	0.087	17.819	< 0.001
	Rikenellaceae	0.107	0.008	0.033	21.684	< 0.001
	Erysipelotrichaceae	0.004	0.002	0.004	7.088	0.033
	*Family*_XIII	0.006	0.002	0.002	18.923	< 0.001
	Lachnospiraceae	0.102	0.063	0.083	9.453	0.012
	Spirochaetaceae	0.003	0.002	0.004	6.562	0.041
	unclassified_o__*Bacteroidales*	0.007	0.005	0.008	7.546	0.029
	Campylobacteraceae	0.009	0.081	0.022	18.827	< 0.001
	Christensenellaceae	0.040	0.090	0.066	15.705	< 0.001
	Muribaculaceae	0.223	0.489	0.502	17.424	< 0.001
	Ruminococcaceae	0.270	0.184	0.110	13.273	0.002
	Nocardiaceae	0.020	0.001	0.000	17.611	< 0.001
	Saccharimonadaceae	0.004	0.002	0.002	4.433	0.109

Table S3 Results of the Wilcoxon rank-sum test comparing KEGG Metabolism categories (level3) between pikas in the JW and JC groups, and JW and JCS groups.

**Pathway_level2**	**Pathway_level3**	**Level3_description**	**JW vs JC FDR *p***	**JW vs JCS FDR *p***
Amino acid metabolism	ko00330	Arginine and proline metabolism	**< 0.001**	**< 0.001**
Amino acid metabolism	ko00340	Histidine metabolism	**< 0.01**	**< 0.001**
Amino acid metabolism	ko00300	Lysine biosynthesis	0.050	**< 0.01**
Amino acid metabolism	ko00270	Cysteine and methionine metabolism	0.050	**< 0.01**
Amino acid metabolism	ko00400	Phenylalanine, tyrosine and tryptophan biosynthesis	0.201	**< 0.05**
Amino acid metabolism	ko00260	Glycine, serine and threonine metabolism	0.095	0.095
Amino acid metabolism	ko00250	Alanine, aspartate and glutamate metabolism	**< 0.05**	0.268
Carbohydrate metabolism	ko00010	Glycolysis / Gluconeogenesis	**< 0.001**	**< 0.001**
Carbohydrate metabolism	ko00630	Glyoxylate and dicarboxylate metabolism	**< 0.001**	**< 0.01**
Carbohydrate metabolism	ko00051	Fructose and mannose metabolism	**< 0.05**	**< 0.01**
Carbohydrate metabolism	ko00650	Butanoate metabolism	0.060	**< 0.05**
Carbohydrate metabolism	ko00520	Amino sugar and nucleotide sugar metabolism	0.084	0.050
Carbohydrate metabolism	ko00040	Pentose and glucuronate interconversions	0.095	**< 0.05**
Carbohydrate metabolism	ko00052	Galactose metabolism	0.268	0.371
Carbohydrate metabolism	ko00620	Pyruvate metabolism	0.296	0.450
Carbohydrate metabolism	ko00500	Starch and sucrose metabolism	0.624	0.050
Carbohydrate metabolism	ko00030	Pentose phosphate pathway	0.721	0.189
Energy metabolism	ko00680	Methane metabolism	**< 0.05**	0.139
Energy metabolism	ko00910	Nitrogen metabolism	**< 0.05**	**< 0.001**
Energy metabolism	ko00710	Carbon fixation in photosynthetic organisms	0.050	**< 0.001**
Energy metabolism	ko00190	Oxidative phosphorylation	0.060	**< 0.05**
Energy metabolism	ko00720	Carbon fixation pathways in prokaryotes	0.180	0.826

Glycan biosynthesis and metabolism	ko00550	Peptidoglycan biosynthesis	0.084	0.968
Glycan biosynthesis and metabolism	ko00511	Other glycan degradation	0.577	0.732
Lipid metabolism	ko00061	Fatty acid biosynthesis	0.224	0.050
Lipid metabolism	ko00564	Glycerophospholipid metabolism	0.268	0.405
Metabolism of cofactors and vitamins	ko00770	Pantothenate and CoA biosynthesis	**< 0.001**	0.050
Metabolism of cofactors and vitamins	ko00860	Porphyrin and chlorophyll metabolism	**< 0.05**	0.732
Metabolism of cofactors and vitamins	ko00730	Thiamine metabolism	0.224	0.545
Metabolism of cofactors and vitamins	ko00790	Folate biosynthesis	0.296	**< 0.05**
Metabolism of cofactors and vitamins	ko00760	Nicotinate and nicotinamide metabolism	0.735	0.268
Metabolism of cofactors and vitamins	ko00670	One carbon pool by folate	0.735	0.968
Metabolism of other amino acids	ko00450	Selenocompound metabolism	0.938	0.701
Metabolism of terpenoids and polyketides	ko00900	Terpenoid backbone biosynthesis	0.405	0.938
Nucleotide metabolism	ko00230	Purine metabolism	**< 0.001**	**< 0.001**
Nucleotide metabolism	ko00240	Pyrimidine metabolism	0.268	0.497

Only those categories with mean relative abundance more than 0.05% are selected. Significant effects are in bold.

Table S4 Results of the Wilcoxon rank-sum test comparing KEGG Metabolism categories (level3) between pikas in the NW and NC groups, and NW and NCS groups.

**Pathway_level2**	**Pathway_level3**	**Level3_description**	**NW vs NC FDR *p***	**NW vs NCS FDR *p***
Amino acid metabolism	ko00260	Glycine, serine and threonine metabolism	**< 0.001**	**< 0.05**
Amino acid metabolism	ko00250	Alanine, aspartate and glutamate metabolism	**< 0.05**	0.050
Amino acid metabolism	ko00300	Lysine biosynthesis	0.120	0.335
Amino acid metabolism	ko00330	Arginine and proline metabolism	0.188	**< 0.05**
Amino acid metabolism	ko00350	Tyrosine metabolism	0.285	**< 0.001**
Amino acid metabolism	ko00400	Phenylalanine, tyrosine and tryptophan biosynthesis	0.285	0.296
Amino acid metabolism	ko00270	Cysteine and methionine metabolism	0.491	0.824
Amino acid metabolism	ko00340	Histidine metabolism	0.724	0.360
Carbohydrate metabolism	ko00620	Pyruvate metabolism	**< 0.001**	**< 0.001**
Carbohydrate metabolism	ko00010	Glycolysis / Gluconeogenesis	**< 0.001**	**< 0.001**
Carbohydrate metabolism	ko00650	Butanoate metabolism	**< 0.01**	**< 0.01**
Carbohydrate metabolism	ko00500	Starch and sucrose metabolism	**< 0.05**	0.360
Carbohydrate metabolism	ko00040	Pentose and glucuronate interconversions	**< 0.05**	0.360
Carbohydrate metabolism	ko00052	Galactose metabolism	0.120	**< 0.05**
Carbohydrate metabolism	ko00051	Fructose and mannose metabolism	0.188	**< 0.05**
Carbohydrate metabolism	ko00630	Glyoxylate and dicarboxylate metabolism	0.215	0.761
Carbohydrate metabolism	ko00030	Pentose phosphate pathway	0.342	0.117
Carbohydrate metabolism	ko00520	Amino sugar and nucleotide sugar metabolism	0.360	**< 0.05**
Energy metabolism	ko00190	Oxidative phosphorylation	**< 0.001**	0.232
Energy metabolism	ko00720	Carbon fixation pathways in prokaryotes	0.077	**< 0.01**
Energy metabolism	ko00680	Methane metabolism	0.586	0.064
Energy metabolism	ko00910	Nitrogen metabolism	0.622	**< 0.001**

Glycan biosynthesis and metabolism	ko00550	Peptidoglycan biosynthesis	**< 0.01**	**< 0.01**
Glycan biosynthesis and metabolism	ko00511	Other glycan degradation	**< 0.05**	0.050
Lipid metabolism	ko00061	Fatty acid biosynthesis	**< 0.001**	**< 0.01**
Lipid metabolism	ko00564	Glycerophospholipid metabolism	0.262	0.050
Metabolism of cofactors and vitamins	ko00730	Thiamine metabolism	**< 0.001**	**< 0.01**
Metabolism of cofactors and vitamins	ko00790	Folate biosynthesis	**< 0.001**	1.000
Metabolism of cofactors and vitamins	ko00860	Porphyrin and chlorophyll metabolism	0.065	0.232
Metabolism of cofactors and vitamins	ko00770	Pantothenate and CoA biosynthesis	0.233	**< 0.05**
Metabolism of cofactors and vitamins	ko00670	One carbon pool by folate	0.312	0.117
Metabolism of cofactors and vitamins	ko00760	Nicotinate and nicotinamide metabolism	0.761	**< 0.05**
Metabolism of other amino acids	ko00450	Selenocompound metabolism	**< 0.001**	0.262
Metabolism of terpenoids and polyketides	ko00900	Terpenoid backbone biosynthesis	0.065	**< 0.05**
Nucleotide metabolism	ko00230	Purine metabolism	0.091	**< 0.01**
Nucleotide metabolism	ko00240	Pyrimidine metabolism	0.622	**< 0.05**

Only those categories with mean relative abundance more than 0.05% are selected. Significant effects are in bold.
